# Development and Validation of a Nomogram-Based Prognostic Model to Predict High Blood Pressure in Children and Adolescents—Findings From 342,736 Individuals in China

**DOI:** 10.3389/fcvm.2022.884508

**Published:** 2022-06-23

**Authors:** Jing-Hong Liang, Yu Zhao, Yi-Can Chen, Shan Huang, Shu-Xin Zhang, Nan Jiang, Aerziguli Kakaer, Ya-Jun Chen

**Affiliations:** Department of Maternal and Child Health, School of Public Health, Sun Yat-sen University, Guangzhou, China

**Keywords:** risk classification, nomogram, children and adolescents, high blood pressure, cross-sectional study

## Abstract

**Objectives:**

Predicting the potential risk factors of high blood pressure (HBP) among children and adolescents is still a knowledge gap. Our study aimed to establish and validate a nomogram-based model for identifying youths at risk of developing HBP.

**Methods:**

HBP was defined as systolic blood pressure or diastolic blood pressure above the 95th percentile, using age, gender, and height-specific cut-off points. Penalized regression with Lasso was used to identify the strongest predictors of HBP. Internal validation was conducted by a 5-fold cross-validation and bootstrapping approach. The predictive variables and the advanced nomogram plot were identified by conducting univariate and multivariate logistic regression analyses. A nomogram was constructed by a training group comprised of 239,546 (69.9%) participants and subsequently validated by an external group with 103,190 (30.1%) participants.

**Results:**

Of 342,736 children and adolescents, 55,480 (16.2%) youths were identified with HBP with mean age 11.51 ± 1.45 years and 183,487 were boys (53.5%). Nine significant relevant predictors were identified including: age, gender, weight status, birth weight, breastfeeding, gestational hypertension, family history of obesity and hypertension, and physical activity. Acceptable discrimination [area under the receiver operating characteristic curve (AUC): 0.742 (development group), 0.740 (validation group)] and good calibration (Hosmer and Lemeshow statistics, *P* > 0.05) were observed in our models. An available web-based nomogram was built online on https://hbpnomogram.shinyapps.io/Dyn_Nomo_HBP/.

**Conclusions:**

This model composed of age, gender, early life factors, family history of the disease, and lifestyle factors may predict the risk of HBP among youths, which has developed a promising nomogram that may aid in more accurately identifying HBP among youths in primary care.

## Introduction

High blood pressure (HBP), whether primary or secondary, occurs with 2–3% incidence in the general children population (1, 2) and is deemed the most common and potentially reversible early marker for the development of metabolic diseases, neurological disorders, persistent hypertension in adulthood, and cardiovascular disease in particular (3, 4), which contributes not only to global burden but is also a major cause of poorer disability-adjusted life-years both for youths and adults (5, 6). It is a controversial fact that boys are more susceptible to the development of primary hypertension especially in China (7). This gender disparity may result from the significantly higher prevalence of obesity in boys compared to girls (8, 9). However, 74% of children that have been diagnosed with high blood pressure (BP) have been underrecognized as hypertensive (10), of which the underlying reasons are not fully understood but are likely due to the asymptomatic presentation of hypertension, challenging patient assessments, complicated BP norms, and unclear recommendations.

Given the increasing evidence base, recommendations of identification regarding BP in children from multiple guidelines vary somewhat (11–14), probably attributed to differences in their region, sample size, and other extrapolations of evidence. Based on pediatric research, it is essential to identify inherent and exterior predictors that may provide comprehensive strategies for unraveling the pathogenesis of hypertension at individuals' early stages.

And another concern is that previous studies yielded contradictory results on the risk factors for HBP in children. For example, some studies indicated that childhood exposure to smoking, whether active or passive, was associated with an increased risk of HBP (15–17). At the same time, some reported inconsistent evidence (18, 19), and a meta-analysis has produced unconvincing evidence from relatively low-quality epidemiologic studies (20). Furthermore, the previous predictive model remains inconsistent. A study performed in Switzerland identified a higher proportion of children with HBP (65%) based on the child being overweight and parental HBP than another strategy with just the child being overweight (43%) (21, 22). Thus, a more plausible model to target screening for children and adolescents with HBP needs to be addressed appropriately.

Consequently, in this study, based on the ongoing successive sectional study conducted in Guangzhou [(Guangzhou Survey on Students' Constitution and Health), GSSCH] in 2020 with a total of 1,594,048 participants, our study aims at assessing the association of numerous predictions and HBP, and to build the first-ever statistical nomogram tool to identify the precise long-term effect modifiers of HBP in children and adolescents both by developing and internally validating the dataset.

## Methods

### Study Population and Data Collection

The survey is not publicly available and participants were protected under a certificate of confidentiality issued by the Government of Guangzhou due to the sensitive nature of data collected from all student groups in Guangzhou city.

Our study was performed in accordance with the Strengthening the Reporting of Observational Studies in Epidemiology (STROBE) reporting guidelines (23). Relevant information was collected from all participants' parents or their legal guardians, who also gave written informed consent which was collected according to the guidelines of the Declaration of Helsinki (24).

This survey was approved by the Ethics and Human Subject Committee of Sun Yat-sen University and any uncertainties were corrected before the consent forms were signed. Data were obtained from the GSSCH, a sequential cross-sectional investigation carried out annually, comprising approximately 1,600 primary and middle schools in Guangzhou city, which was completed and supported by Sun-Yat Sen University, Guangzhou school-health promotion center, and Guangzhou Education Bureau. The GSSCH has been conducted for 4 consecutive years, and a battery of relevant measures was undertaken during September and October, the first 2 months of a new school year.

### Collecting Data and Definition of Covariates

The dataset was independently divided by the cross-sectional study conducted in 2020 for identifying the first-ever incidence of HBP among children and adolescents. All participants were students aged from 5 to 25 years old, and trained investigators or practitioners conducted questionnaires on health-related behaviors, administered regular physical examinations, and collected objective anthropometric data measurements. A total of 1,102,462 children were removed due to missing data, incomplete data, or extreme values respecting the demographic variables or relevant BP variables based on the four sex and age-specific standard deviations (SDs). Overall, 148,850 participants were aged above 18. And a total of 342,736 subjects were included in our analysis after the eliminations mentioned above. The subject group was split into training and validation sets by taking a random sample of 69.9% (*N* = 239,546) for training and the remaining 30.1% (*N* = 103,190) for validation.

Variable information comprising demographic characteristics (e.g., age, sex, family income), lifestyle-associated factors [e.g., physical activity (PA), dietary pattern, sleep pattern], and health-related information (including medical history, past, current diseases, and family history) for HBP was assessed in 2020 as the training cohort and validation database, through matching individuals' identifications. Body mass index (BMI) was calculated as the individual's body weight in kilograms divided by the square of height in meters and further divided into four categories (underweight, average weight, overweight, obese) adjusted for age and gender. Parental education level was defined as the highest level of education reported for either parents, divided into primary school or below, secondary school, senior high school or junior school, junior college or university, and graduate or above. Parental smoking status separately for the father and mother was combined with three categories: never smoked (both parents had never smoked), and former or current smokers (either of the parents were former or current smokers). The family history of obesity and hypertension was defined by the reported history of obesity and hypertension in immediate family members, while gestational hypertension was defined as new-onset hypertension after 20 weeks gestation.

### Measurement of High Blood Pressure

The children were asked to sit comfortably for at least 10 min before BP measurement, which was conducted in a quiet area. A standard BP cuff was positioned near the right elbow, 2 cm below the antecubital fossa. BP for each child was recorded three consecutive times in a seated position on their right arm by a calibrated electronic sphygmomanometer (OMRON, HEM-4001C, Kyoto, Japan) on a single occasion. The diagnosis of HBP was in accordance with the updated BP diagnostic criteria for Chinese children and adolescents that have an average systolic blood pressure (SBP) or diastolic blood pressure (DBP) ≥95th percentile for the corresponding child's age, sex, and height (25).

### Statistical Analyses

The continuous variables of participants were presented as mean with standard deviation and interquartile range (IQR), whereas categorical data were revealed as absolute frequencies with percentages. For differences between groups of continuous and categorical variables, the two-sample *t*-test or Mann-Whitney test and Chi-Square test were applied, as appropriate. We first used least absolute shrinkage and selection operator (Lasso)-penalized logistic regression to identify significant predictors by presenting the significance with α = 0.05 with over 1,000 bootstrap iterations (26). The odds ratio (OR) with the following 95% confident interval (CI) was computed. Multivariable logistic regression was generated by using consecutive candidate variable factors that discarded predictors according to the *P*-values in a stepwise manner (27). Along with the nomogram, predicted probability of HBP was constructed. The Bayesian information criterion and Akaike information criterion (AIC) were used to evaluate the maximum likelihood model respecting multivariable logistic regression. Moreover, we constructed the area under the receiver operating characteristic curve (AUC, or C-index) to assess the model discrimination. In contrast, the Hosmer-Lemeshow approach was used and a calibration curve was built for detecting model calibration. Here, all analyses were conducted using R language (X64 Version 4.1.0, R Foundation for Statistical Computing, Vienna, Austria, https://www.r-project.org/) with several packages including “Foreign,” “Hmisc,” “Glmnet,” “Caret,” “Rms,” and “pROC.” The bilateral *P* < 0.05 was recognized as statistically significant.

## Results

### Demographic Characteristics

As displayed in Supplementary Figure 1, based on the several screening criteria, a total of 342,736 participants were identified after exclusions. A total of 69.9% (*N* = 239,546) of students were randomly assigned to the derivation group and 30.1% (*N* = 103,190) to the internal validation group. Of the development group, the average age of children and adolescents was 11.51 years (2.73) with a range from 6 to 18, and 11.51 years (2.74) with a range from 7 to 18 in the validation group. More than half of the children were boys (53.5%, *n* = 183,487). Of 342,736 participants, 287,256 (83.8%) and 55,480 (16.2%) children were identified as having non-HBP and HBP, respectively.

There was a statistically significant difference among the majority of demographic variables in either the HBP group or non-HBP group (*P* < 0.001), such as age, gender, birth weight, and so on. We found no significant differences in one parental education level only. The demographic characteristics of the subjects are listed in Table 1.

**Table 1:** Comparison of baseline characteristics between HBP group and non-HBP group.

**Characteristics**	**Total (*n*= 342,736)**	**HBP group (*n* = 55,480)**	**Non-HBP group (*n* = 287,256)**	***P*** **-value**
**Age (year) (mean ±SD)**	11.51 ± 2.73	12.22 ± 2.88	11.37 ± 2.68	<0.001
**Boys [*****n*** **(%)]**	183,487 (53.5)	35,246 (63.5)	148,241 (51.6)	<0.001
**Feeding mode [*****n*** **(%)]**				<0.001
Breastfeeding	136,442 (39.8)	21,455 (38.7)	114,987 (40.0)	
Bottle feeding	140,342 (40.9)	18,325 (33.0)	122,017 (42.5)	
Partial breastfeeding	65,952 (19.2)	15,700 (28.3)	50,252 (17.5)	
**Birthweight (g) (mean ±SD)**				<0.001
2,500–4,000	307,250 (89.6)	40,922 (73.8)	266,328 (92.7)	
<2,500	34,046 (9.9)	14,117 (25.4)	19,929 (6.9)	
>4,000	1,440 (0.4)	441 (0.8)	999 (0.3)	
**Gestational hypertension [*****n*** **(%)]**	6,133 (1.8)	1,355 (2.4)	4,778 (1.7)	<0.001
SBP (mmHg)	104.05 ± 10.17	116.10 ± 8.25	101.73 ± 8.76	<0.001
DBP (mmHg)	64.95 ± 6.88	71.17 ± 6.48	63.75 ± 6.29	<0.001
BMI (kg/m2) (mean ± SD)	18.00 ± 3.36	19.25 ± 3.88	17.75 ± 3.19	<0.001
**Weight status [*****n*** **(%)]**				<0.001
Underweight	39,382 (11.5)	8,682 (15.6)	30,700 (10.7)	
Normal weight	241,1212 (70.4)	36,145 (65.1)	205,067 (71.4)	
Overweight	26,443 (7.7)	6,749 (12.2)	19,694 (6.9)	
Obese	35,699 (10.4)	3,904 (7.0)	31,795 (11.1)	
**Parental smoking status [*****n*** **(%)]**				<0.001
Never smokers	156,156 (45.6)	25,208 (45.4)	130,948 (45.6)	
Former smokers	14,839 (12.2)	6,987 (12.6)	34,852 (12.1)	
Current smokers	144,741 (42.2)	23,285 (42.0)	121,456 (42.3)	
**Parental education level [*****n*** **(%)]**				0.552
Primary school or below	5,194 (1.5)	866 (1.6)	4,328 (1.5)	
Secondary school	87,255 (25.5)	14,085 (25.4)	73,170 (25.5)	
Senior high school or junior school	93,230 (27.2)	15,023 (27.1)	78,207 (27.2)	
Junior college or university	142,442 (41.6)	23,084 (41.6)	119,358 (41.6)	
Graduate or above	14,615 (4.3)	2,422 (4.4)	12,193 (4.2)	
**Family history of hypertension [*****n*** **(%)]**	126,981 (37.0)	12,593 (22.7)	114,388 (39.8)	<0.001
**Family history of obesity [*****n*** **(%)]**	48,135 (14.0)	8,295 (15.0)	39,840 (13.9)	<0.001
**Household monthly income [*****n*** **(%)]**				<0.001
<5,000, RMB	156,994 (45.8)	24,547 (44.2)	132,447 (46.1)	
5,000–7,999, RMB	78,761 (23.0)	13,161 (23.7)	65,600 (22.8)	
8,000–11,999, RMB	69,654 (20.3)	11,657 (21.0)	57,997 (20.2)	
≥12,000, RMB	37,327 (10.9)	6,115 (11.0)	31,212 (10.9)	
**PA time (Mins/day) (mean ±SD)**	62.03 ± 53.64	62.63 ± 54.22	61.92 ± 53.53	0.004
**Average outdoor physical activity time [*****n*** **(%)]**				<0.001
<1 h/day	157,116 (45.8)	29,274 (52.8)	127,842 (44.5)	
1–1.9 h/day	151,117 (44.1)	20,813 (37.5)	130,304 (45.4)	
2–4 h/day	29,311 (8.6)	4,534 (8.2)	24,777 (8.6)	
>4 h/day	5,192 (1.5)	859 (1.5)	4,333 (1.5)	
**SBT (Mins/day) (mean ±SD)**	41.97 ± 47.85	44.10 ± 51.37	41.56 ± 47.13	<0.001
**Average Screen-based time [*****n*** **(%)]**				<0.001
<2 h/day	324,517 (94.7)	52,030 (93.8)	272,487 (94.9)	
2–4 h/day	14,861 (4.3)	2,761 (5.0)	12,100 (4.2)	
>4 h/day	3,358 (1.0)	689 (1.2)	2,669 (0.9)	
**Fried food intake (serving/week)**	0.89 ± 1.18	0.91 ± 1.23	0.88 ± 1.17	<0.001

*Data were presented as mean (SD), median (IQR) or n (%). BMI, Body mass index; HBP, High blood pressure; PA, Physical activity; RMB, Renminbi/Yuan; SBT, Screen-based time; SDP, Systolic blood pressure; DBP, Diastolic blood pressure*.

The prevalence of HBP was 16.19% [development group: 16.20% (*n* = 38,807), validation group: 16.16% (*n* = 16,673)] judged by the 95th percentile for gender, age, and height. The mean SBP and DBP were 104.10 ± 10.17 mmHg and 64.95 ± 6.88 mmHg in total participants.

### Multivariable Predictors of HBP

**Figure 1:**
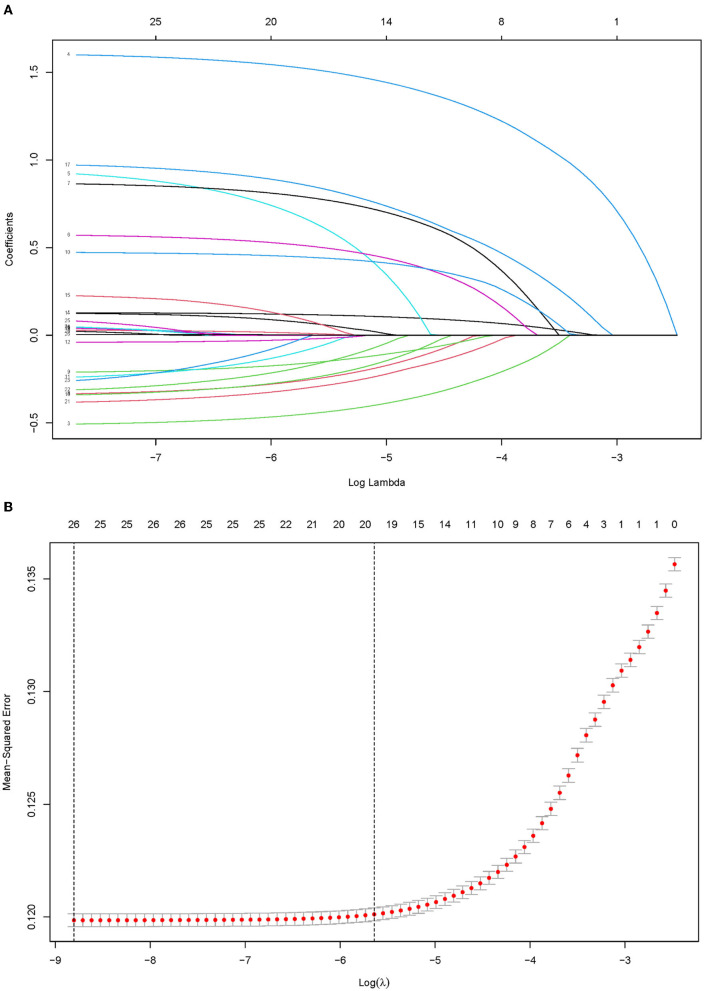
Predictor selection using the Lasso binary logistic regression model. A Lasso coefficient of the total 14 predictors. **(A)** Lasso coefficient profiles of all predictors, a coefficient profile plot was provided against the log (Lambda) sequence. **(B)** Predictors selection by Lasso *via* minimum criteria, predictor selection in the Lasso model used 10-fold cross-validation *via* minimum criteria. Red-dotted vertical lines were drawn at the optimal values by using the minimum criteria (minimizing the mean-squared error), the value 9 represents that those 14 predictors were reduced to 9 non-zero features by Lasso.

According to the pre-established preoperative variables evaluated, Lasso regression showed that there were 14 candidate predictors including age, gender, birth weight, weight status, feeding mode, gestational hypertension, parental smoking status, parental education, family history of obesity, family history of hypertension, monthly household income, average outdoor physical activity time, average screen-based time, and fried food intake. The incidence of HBP for children and adolescents (shown in Figure 1), highly correlated in only nine of them (age, gender, birth weight, weight status, feeding mode, gestational hypertension, family history of obesity, family history of hypertension, and average outdoor physical activity time), was independently associated with a higher probability of HBP after the backward elimination procedure of multivariable logistic analysis. Table 2 shows the results of multivariable predictors.

**Table 2:** Multivariable Logistic regression analysis of variables predicting HBP in the development Group.

**Variables**		**OR**	***P*** **-value**
Age		1.142 (1.137–1.147)	<0.001
**Gender**			
Boy	Ref		
Girl		0.593 (0.578–0.607)	<0.001
**Feeding mode**			
Breastfeeding	Ref		
Bottle feeding		1.656 (1.608–1.706)	<0.001
Partial breastfeeding		0.833 (0.811–0.856)	<0.001
**Birthweight**			
2,500–4,000	Ref		
<2,500		4.906 (4.757–5.060)	<0.001
>4,000		2.494 (2.157–2.876)	<0.001
**Gestational hypertension**			
Gestational hypertension	Ref		
Non-Gestational hypertension		0.720 (0.665–0.779)	<0.001
**Weight status**			
Underweight	Ref		
Normal weight		0.715 (0.684–0.747)	<0.001
Overweight		1.825 (1.765–1.888)	<0.001
Obese		2.430 (2.337–2.526)	<0.001
**Parental smoking status**			
Never smokers	Ref		
Former smokers		1.020 (0.982–1.059)	0.301
Current smokers		1.012 (0.987–1.038)	0.362
**Parental education level**			
Primary school or below	Ref		
Secondary school		1.132 (1.029–1.247)	0.012
Senior high school or junior school		1.188 (1.080–1.310)	<0.001
Junior college or university		1.352 (1.229–1.490)	<0.001
Graduate or above		1.521 (1.361–1.701)	<0.001
**Family history of hypertension**			
Non-Family history of hypertension	Ref		
Family history of hypertension		2.618 (2.545–2.693)	<0.001
**Family history of obesity**			
Family history of obesity	Ref		
Non-Family history of obesity		0.706 (0.682–0.731)	<0.001
**Household monthly income**			
<5,000,RMB	Ref		
5,000–7,999, RMB		1.058 (1.026–1.090)	<0.001
8,000–11,999, RMB		1.045 (1.011–1.079)	<0.001
≥12,000, RMB		1.055 (1.012–1.100)	0.011
**Average outdoor physical activity time**			
<1 h/day	Ref		
1–1.9 h/day		0.672 (0.655–0.689)	<0.001
2–4 h/day		0.724 (0.693–0.756)	<0.001
>4 h/day		0.755 (0.687–0.829)	<0.001
**Average Screen-based time**			
<2 h/day	Ref		
2–4 h/day		1.053 (0.996–1.113)	0.066
>4 h/day		1.191 (1.069–1.325)	<0.001
**Fried food intake (serving/week)**		1.035 (1.026–1.045)	<0.001

*BMI, Body mass index; HBP, High blood pressure; OR, Odd ratio; PA, Physical activity; RMB, Renminbi/Yuan; SBP, Systolic blood pressure; DBP, Diastolic blood pressure*.

**Figure 2:**
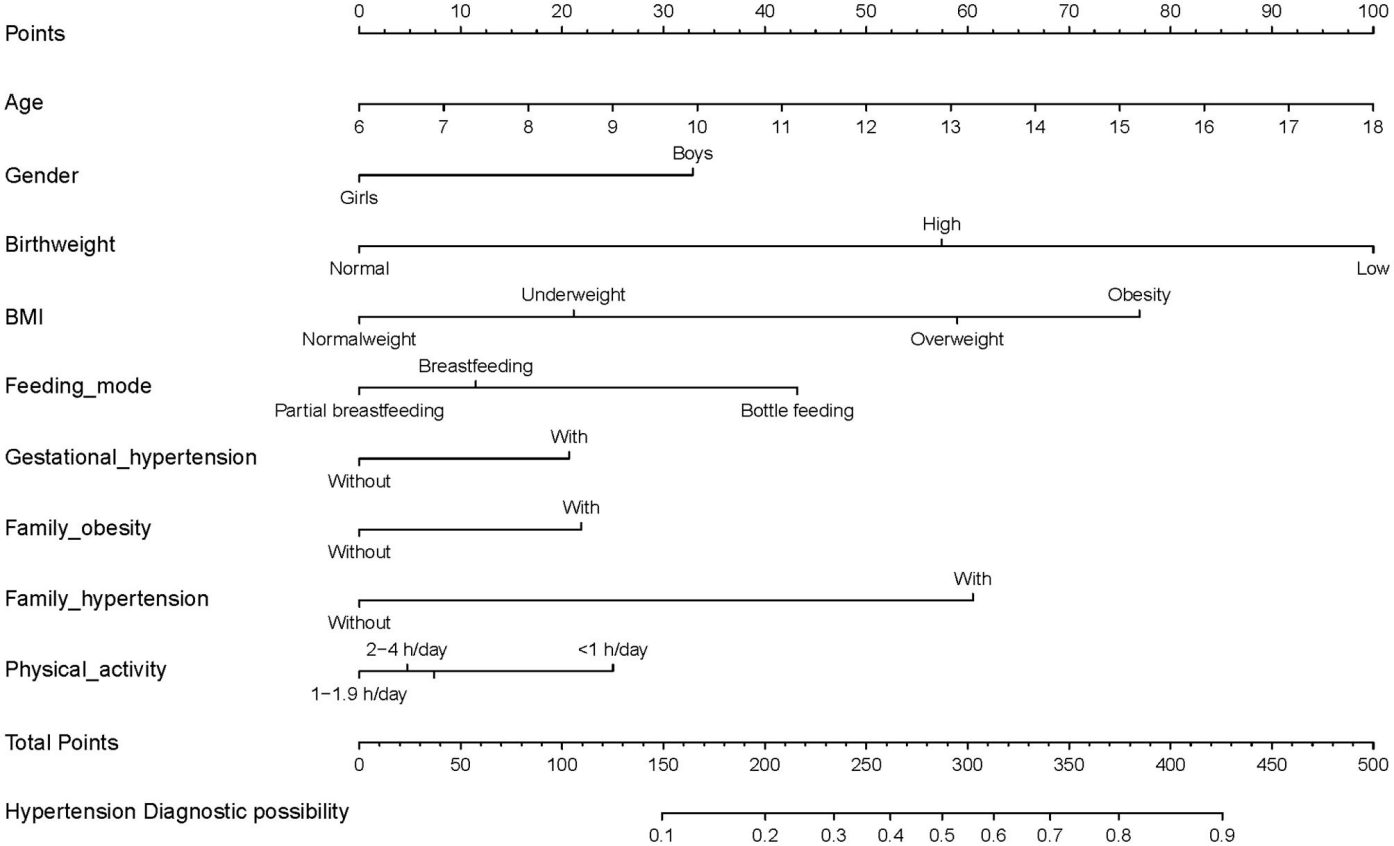
Clinical nomogram for predicting probability of developing HBP among children and adolescents, and its predictive performance. To use the nomogram, an individual HBP contact's values are located on each variable axis, and a line is drawn downward to the risk of HBP axes to detect the hypertension probability. As an example of how this nomogram can be calculated, we can take an 18-year-old obese boy who was bottle-fed in early life, with gestational hypertension, family history of hypertension and obesity, and <1-h physical activity expenditure. By drawing a line up toward the points for each of the variables this student will have 100 points (age), 32 points (gender), 74 points (BMI status), 42 points (feeding mode), 20 points (gestational hypertension), 21 points (family obesity), 59 points (family hypertension), and 26 points (physical activity), giving a total of 374 points (at the bottom of the figure), and a probability of HBP of 80%.

**Figure 3:**
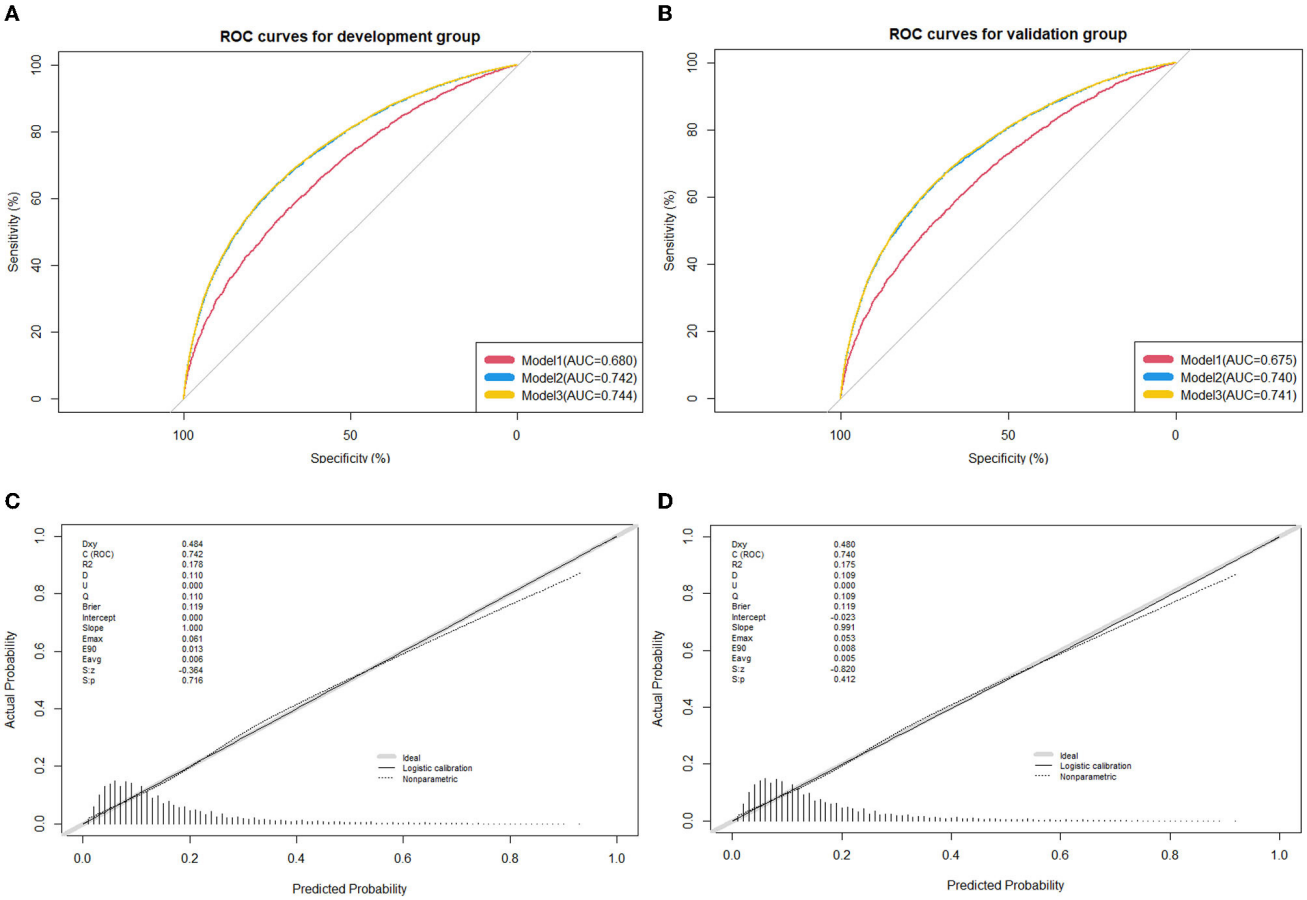
Receiver operating characteristic (ROC) curves for the prediction of high blood pressure in the training group and validation group. **(A)** ROC curves of the factors and nomogram in the development group; **(B)** ROC curves of the factors and nomogram in the training group; **(C)** calibration plot of nomogram prediction in the development group; **(D)** calibration plot of nomogram prediction in the validation group. ROC curves from the prediction model and other predictive strategies (Model 1: Age, gender, gestational hypertension, weight status, family history of hypertension, family history of obesity, and average outdoor physical activity time; Model 2: Age, gender, gestational hypertension, weight status, family history of hypertension, family history of obesity, average outdoor physical activity time, birth weight, and feeding mode; and Model 3: Age, gender, gestational hypertension, weight status, family history of hypertension, family history of obesity, average outdoor physical activity time, birth weight, feeding mode, parental smoking status, parental education level, household monthly income, average screen-based time, and fried food intake) for comparison. The calibration curve represents the calibration of the nomogram, which shows the consistency between the predicted probability of conversion and actual conversion probability of HBP patients. The x-axis is the predicted probability by the nomogram and the y-axis is the actual conversion rate of HBP patients. The gray line represents a perfect prediction by an ideal model, and the black-dotted line shows the performance of the nomogram, of which a closer fit to the gray line means a better prediction.

The nomogram plot based on all nine independent predictors calculated by the generalized linear models is depicted in Figure 2, with a single score of each predictor contributing to a total score for predicting HBP. The model discrimination as per the above nine significant risk factors was detected by the apparent C-statistic for the model with a value of 0.742 in the development group and 0.740 in the validation group (shown in Figure 3). The model included all the 14 candidate factors, and the AUC was 0.744 and 0.741 in the development and validation groups, respectively (shown in Figure 2). All of the abovementioned tests indicated good discrimination, as shown in Figure 3. Decision curve analysis (DCA) presented in Supplementary Figure 3 further suggested that our nomogram yielded a high clinical net benefit, which is of great value for the accurate, individualized measurement of the incidence of HBP among children and adolescents.

### Website of Nomogram

An available web-based nomogram calculator of HBP was built (https://hbpnomogram.shinyapps.io/Dyn_Nomo_HBP/) to present the diagnostic probability for helping guardians and physicians to identify HBP among children and adolescents in a user-friendly way.

## Discussion

As high potential hazard predictors reported previously, age, gender, birth weight, feeding mode, gestational hypertension, weight status, family history of obesity, family history of hypertension, and frequency of PA were also found and presented as a simple and reliable nomogram plot which was designed for identifying children and adolescents who were at high risk of developing HBP.

Accumulating evidence supports the view that the root contributions of essential hypertension extend back to childhood and adolescence (28, 29). Targeted identification of the risk factors of developing HBP in children and adolescents may be practicable to avoid unnecessary burden, overdiagnosis, or costs. Our study provides more interpretability and more precise classification standards for HBP in youths, instead of using a complicated model to predict HBP.

The most potent risk factor for primary HBP in children and adolescents is elevated body mass index (19), also demonstrated in our study. A higher prevalence in children with HBP who were classified as overweight was observed in the comprehensive meta-analysis (30). Similar prevalence values were also noted in previous studies conducted in America and Europe suggesting that the incidence of HBP among children and adolescents with obesity significantly increased compared to non-obese children (2, 31). There are several known pathophysiological pathways by which obesity may lead to an elevated BP and HBP. HBP often coincides with dysfunctional adipocytes and neurohormonal activation of the sympathetic nervous system (SNS). Increased SNS activity can lead to elevated BP and HBP by increasing renin–angiotensin–aldosterone system (RAAS) activity in addition to the direct vasoconstricting effects of SNS. RAAS activity increases BP directly (angiotensin II-mediated vasoconstriction and further SNS activation) and indirectly (angiotensin II- and aldosterone-mediated salt and water tubular reabsorption and ADH-mediated water retention) (32, 33). Additionally, in an obese kidney, there is increased renal interstitial fluid pressure and slower tubular flow rates which ultimately leads to increased sodium reabsorption and increased intravascular volume (34), which may cause hypertension. Evidence is accumulating that factors during the early-life stage have long-term effects on later BP in childhood and young adulthood (35, 36). As the indicator operating early in life, history of low or high birth weight (birth weight <2,500 g or >4,000 g) seemed to be associated with a higher risk of HBP (37, 38). Nevertheless, some studies did not support this association (39). With regard to another modifiable environmental factors, previous studies have corroborated the protective role of breastfeeding on BP later in life (40, 41), on the contrary, our model indicated that compared with breastfeeding mode, partial breastfeeding may play a protective role while bottle feeding may be considered to be a risk to individuals. Another meta-analysis indicates that any effect of breastfeeding on BP is modest with limited public health importance partly attributed to the publication bias (42). Notwithstanding the fact that early-life factors had possible associations with increased risk for HBP among youths is inconclusive, their long-term efficacy raises concerns and further investigation is desirable.

Our findings, which were consistent with the majority of studies, revealed that other risk factors increased the risk of HBP, including male sex, family history of hypertension, and insufficient exercise (21, 43, 44). Our model shows that lower levels of PA are correlated with a higher risk of HBP, and other studies have commonly shown a beneficial role of PA for youths with HBP (45). Moreover, our finding also suggested that an excess of SB is associated with an increased risk of hypertension compared to the recommended levels of SB. Overall, efforts to encourage youths to increase their PA, and reduce their time of SB are warranted. Based on the initial regression shrinkage and selection of Lasso for fried food intake, there were no statistically significant differences in youths for HBP. This discrepancy could be linked to the fact that salt intake in this study was not included due to the lack of data, even though we roughly use fried food instead of salt intake.

Previous studies using a similar methodology supported the hypothesis that age, gender, birth weight, family history of HBP, and other predictors may account for the high prevalence of HBP during childhood and adolescence. Nevertheless, other models suggested that child ethnicity and environmental and genetic risk factors play critical roles in the onset and progression of HBP (21, 46). It should be noted that the regional differences, eating habits, and differences in morphometric characteristics can explain the dissimilar findings between studies (47). Of the examined risk factors, both age and gender are important contributions to the progression of HBP, and may be more significant if other modifiable risk factors were involved in their life, which is particularly true for essential hypertension (48). Since dichotomization of continuous variables may cause loss of valuable information (49, 50), we have categorized continuous predictors such as PA and SB, especially weight status (BMI), because of normal changes in BMI that occur as children age (19).

To date, our study has established a quantitative nomogram for the first time in the form of a concise, more practical, policy making-orientated HBP nomogram for children that is not only useful for clinicians, public health practitioners, and policymakers in developed countries to address this global health need among various indications but also, and perhaps more importantly, for those in low-income countries. In the macro-view, identifying the HBP predictors for youths would relieve the burden of monitoring and administering youth health and economizing facilities and resources. Furthermore, such identifications can help introduce earlier interventions for those at risk, which include, but are not limited to, helping children disengage themselves from the concern of diseases as early as possible and preventing the transfer to other potential hazards such as adverse cardiovascular event outcomes. From the methodological point of view, numerous selective variables were incorporated into a comprehensive instrument to predict the probability of new high blood disorder among youths. Selected summary variables were based on the individuals' demographic characteristics, family history, physical examination, and lifestyle-associated factors. To improve the approach to avoid the concerns of model overfitting, the predictive value of the nomogram is easy to handle, and was evaluated based on highly discrimination, calibration, and clinical utility in separate internal training and validation datasets. One recent publication has developed and validated a risk prediction model for screening hypertension among Chinese adults (51). Targeting this large-scale study on the youth population, our study suggests that nine predictors are significantly associated with HBP among youths. All predictors are readily available in routine physical examinations and easily measured by individuals themselves, which means the established model in our study represents a possibly useful instrument for rapid assessment of HBP among children and adolescents compared with the former model. Furthermore, our study has more advantages than other prediction models as it is based on the youth population with higher discrimination, larger sample size, and more selective candidates for simultaneous estimation (21). As for the diagnostic criteria of HBP, for defining suitable criteria for HBP diagnosis in a later study, additional national evidence is necessary for comparison to confirm their applicability such as the national definition calculated by Dong (52). The Corona Virus Disease 2019 (COVID-19) pandemic has had a tremendous impact on pulmonary hypertension (PH) among youths, from diagnosis to management (53, 54). Nevertheless, it is unknown if PH patients are at a higher risk of developing COVID-19, while there is strong evidence to indicate that youths with PH are more likely to contract pneumonia or other infectious complications alongside COVID-19 (55). The long-term consequences of COVID-19 on cardiovascular events in youths are largely unclear, and primary care clinicians need to seek information about diagnosis and management of HBP for youths to fully optimize the strategy against COVID-19 in children and adolescents with HPB.

However, several limitations exist in the present study. We developed a nomogram model for estimating the risk of HBP while it was validated with an internal assessment dataset. The incidence of HBP (16.9%) in our study was higher than the reported incidence in the general youth population (4.0%) (30), which may have biased our results. More characteristics of patients and variables should be established and extracted to improve the capacity of predicting prognosis in youths with HBP; both genetic and environmental determinants are likely to be responsible for HBP occurrence (43).

## Conclusions

In this study, a feasible nomogram was derived from the ongoing study to visually predict the probability of HBP in children and adolescents for clinicians, policymakers, and even family members to enhance the screening and subsequent diagnosis.

## Data Availability Statement

The raw data supporting the conclusions of this article will be made available by the authors, without undue reservation.

## Ethics Statement

This survey was approved by the Ethics and Human Subject Committee of Sun Yat-sen University. All participants' parents or legal guardian gave written informed consent which was collected according to the guidelines of the Declaration of Helsinki.

## Author Contributions

J-HL conducted the database search, screened and extracted data for the manuscript, and had primary responsibility in writing this article. YZ, Y-CC, SH, S-XZ, NJ, and AK interpretation of data and drafted the initial manuscript and contributed to the discussion and editing. Y-JC supervised data collection and critically edited the final manuscript. All authors approved the final manuscript as submitted, agree to be accountable for all aspects of the work, and read and approved the final manuscript.

## Funding

This work was supported by the National Natural Science Foundation of China (No. 81673193).

## Conflict of Interest

The authors declare that the research was conducted in the absence of any commercial or financial relationships that could be construed as a potential conflict of interest.

## Publisher's Note

All claims expressed in this article are solely those of the authors and do not necessarily represent those of their affiliated organizations, or those of the publisher, the editors and the reviewers. Any product that may be evaluated in this article, or claim that may be made by its manufacturer, is not guaranteed or endorsed by the publisher.
