# Supplementary material for: Development and Validation of a Nomogram-Based Prognostic Model to Predict High Blood Pressure in Children and Adolescents—Findings From 342,736 Individuals in China
Source: Front Cardiovasc Med. 2022 Jun 23;9:884508. doi: 10.3389/fcvm.2022.884508 (PMC9260112; doi:10.3389/fcvm.2022.884508)
Supplement: Supplementary file 1 [file Image_1.pdf]

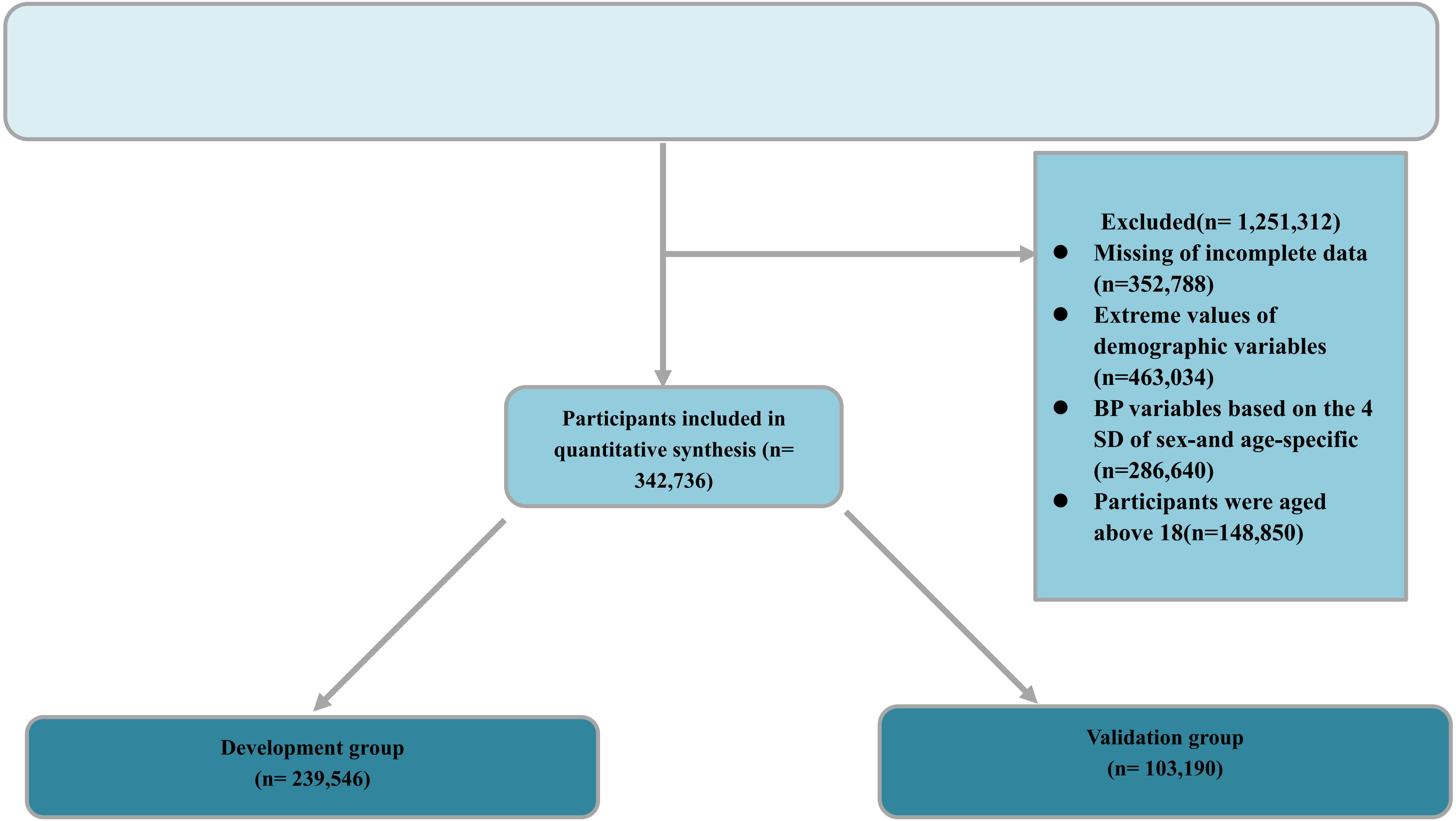

Supplementary Figure 1 Flow chart illustrating the inclusion and exclusion of participants into the study. BP, Blood pressure; HBP, High blood pressure; SD, Standard deviation.
