# Supplementary figures and images for: Development and Validation of a Nomogram-Based Prognostic Model to Predict High Blood Pressure in Children and Adolescents—Findings From 342,736 Individuals in China
Source: Front Cardiovasc Med. 2022 Jun 23;9:884508. doi: 10.3389/fcvm.2022.884508 (PMC9260112; doi:10.3389/fcvm.2022.884508)

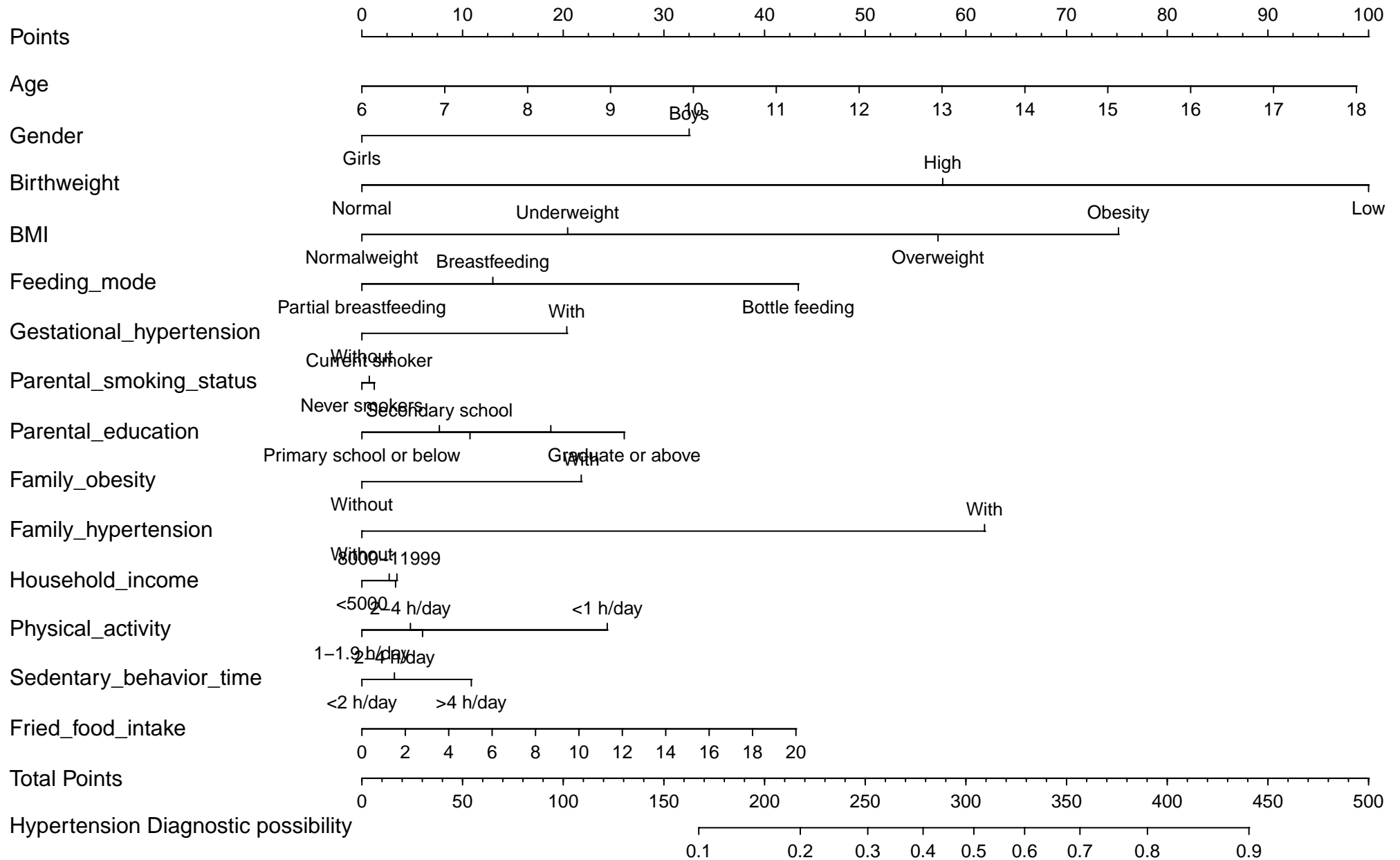

Supplement: Supplementary file 2 [file Image_2.pdf]
