# Supplementary material for: Development and Validation of a Nomogram-Based Prognostic Model to Predict High Blood Pressure in Children and Adolescents—Findings From 342,736 Individuals in China
Source: Front Cardiovasc Med. 2022 Jun 23;9:884508. doi: 10.3389/fcvm.2022.884508 (PMC9260112; doi:10.3389/fcvm.2022.884508)
Supplement: Supplementary file 3 [file Image_3.pdf]

A

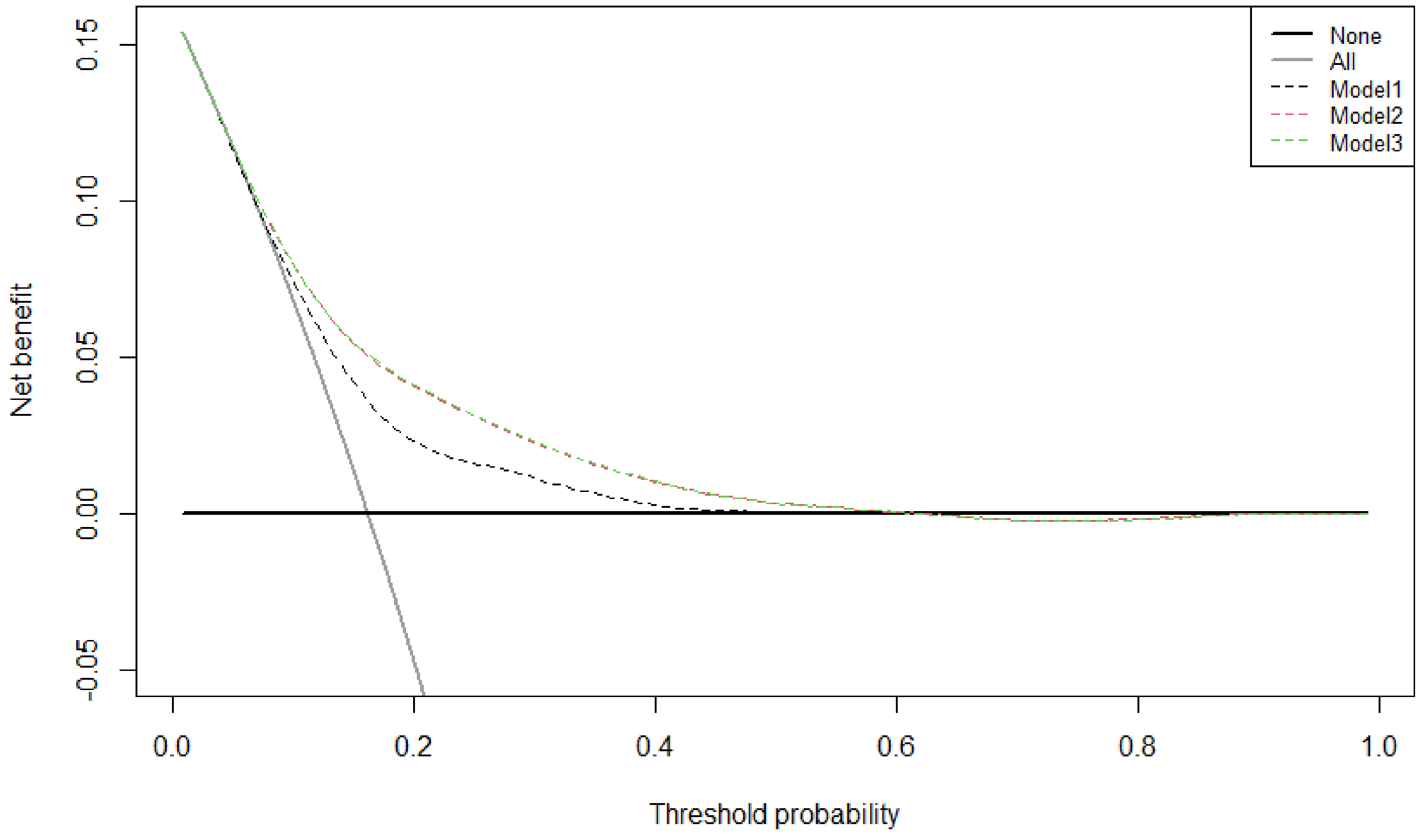

B

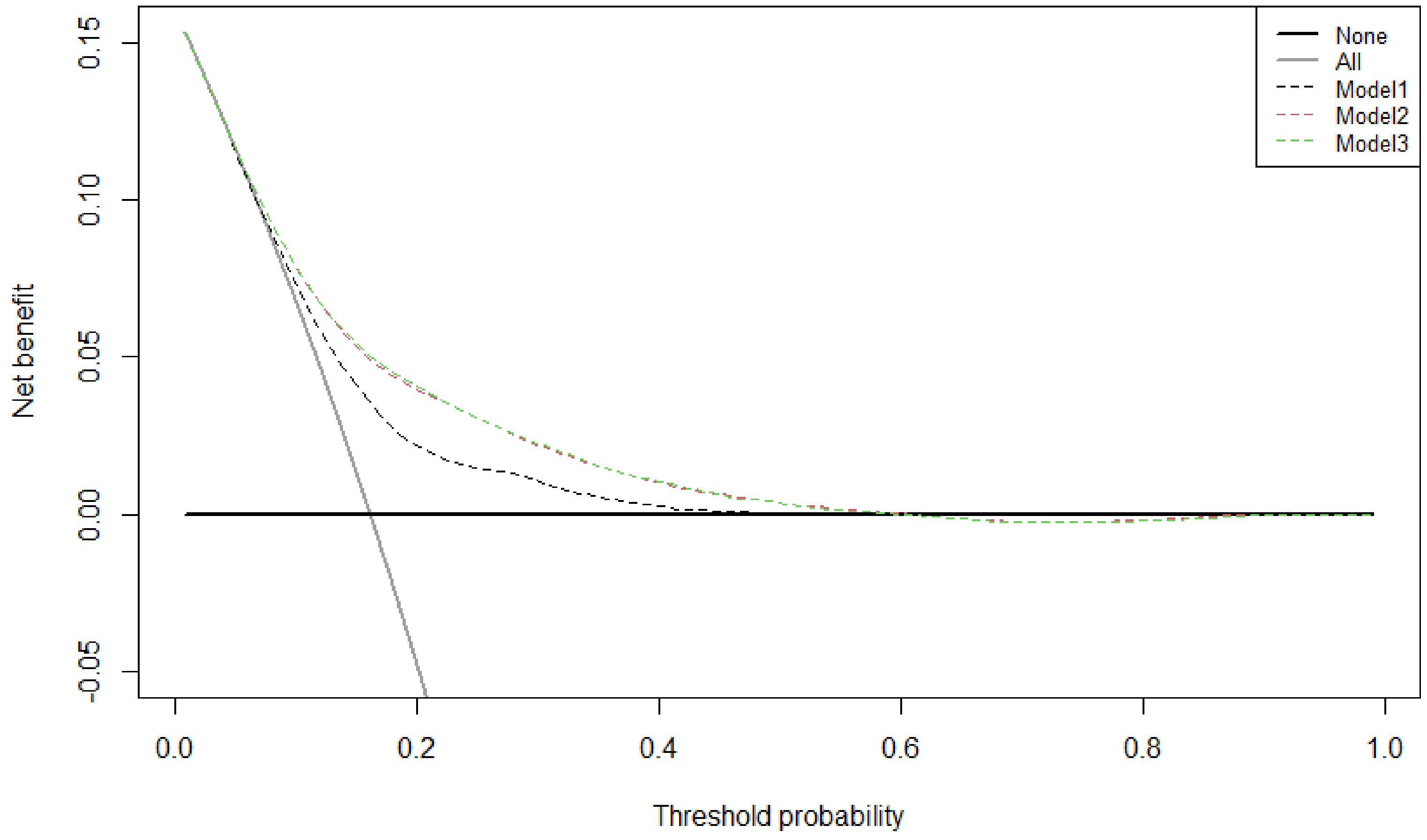

Supplementary Figure 3 Decision curve analysis of the nomogram prediction in the training group and validation group.
(A) Decision curve analysis of the nomogram prediction in the training group; (B) Decision curve analysis of the nomogram prediction in the validation group; Model1:Age, gender, gestational hypertension, weight status, family history of hypertension, family history of obesity, average outdoor physical activity time; Model2: Age, gender, gestational hypertension, weight status, family history of hypertension, family history of obesity, average outdoor physical activity time, birthweight, feeding mode; Model3: Age, gender, gestational hypertension, weight status, family history of hypertension, family history of obesity, average outdoor physical activity time, birthweight, feeding mode; parental smoking status, parental education level, household monthly income, average screen-based time, fried food intake.
